# Comparison Of carcinoembryonic antigen levels between Portal and PERipheral blood in patients with appendiceal adenocarcinoma (COPPER) trial

**DOI:** 10.1093/bjs/znae052

**Published:** 2024-03-08

**Authors:** Adam T Cristaudo, David L Morris, Mohammad Breakeit

**Affiliations:** Liver and Peritonectomy Unit, Department of Surgery, St George Hospital, Kogarah, New South Wales, Australia; Liver and Peritonectomy Unit, Department of Surgery, St George Hospital, Kogarah, New South Wales, Australia; Liver and Peritonectomy Unit, Department of Surgery, St George Hospital, Kogarah, New South Wales, Australia

In 1987, Tabuchi *et al.*^1^ conducted a study to investigate the drainage of carcinoembryonic antigen (CEA) in colorectal cancer, which suggested that CEA is released into the portal blood from primary colonic cancer lesions. Recently, the role of CEA in promoting liver metastases in colorectal cancer has also been highlighted^2^. The authors have conducted a prospective study to test the hypothesis that CEA bypasses the liver and drains via the inferior vena cava in patients with appendiceal adenocarcinoma (AA)^3^. This hypothesis was based on the significant disparity observed in the proportion of liver metastases between AA and colorectal adenocarcinoma^4^.

This study was conducted as a prospective clinical trial and registered in the Australian New Zealand Clinical Trials Registry (ANZCTR) (ACTRN12623000413628). Ethical approval was obtained from the South Eastern Sydney Local Health District Human Research Ethics Committee. The protocol, available on the ANZCTR website, outlines the methodology of the trial.

Sixteen participants from St George Hospital in Sydney, Australia, were recruited prospectively. Peripheral and portal venous blood samples were collected during surgery to compare CEA levels. Sampling was done as described by Tabuchi *et al.*^1^ by Fellows of the Royal Australasian College of Surgeons under the guidance of consultant surgeons.

Median peripheral and portal vein CEA levels were similar, supporting the hypothesis (10 (i.q.r. 5.5–104) and 8.5 (3.0–53) µg/ml respectively; *P* = 0.704). Median patient age was 66 (i.q.r. 52–72) years. One patient (6%) from the cohort died after surgery, but no metastases were observed during the study interval (*Table 1*).

**Table 1:** Baseline characteristics of patients with appendiceal adenocarcinoma

	No. of patients* (*n* = 16)
**Demographics**	
Age (years), median (i.q.r.)	66 (52–72)
Sex	
Male	4 (25)
Female	12 (75)
**Preoperative blood measurements**	
C-reactive protein (mg/l), median (i.q.r.)	8.0 (3.5–23)
α-Fetoprotein (kunits/l), median (i.q.r.)†	2 (2–3)
CA125 (kunits/l), median (i.q.r.)	33 (18–47)
CA19-9 (kunits/l), median (i.q.r.)	14 (9.0–42)
CEA (µg/ml)	10 (5.5–104)
Raised CEA (> 5 ng/ml)	14 (88)
**Operative data**	
ASA fitness grade, median (i.q.r.)	III (III–IV)
Duration of operation (h), median (i.q.r.)	11 (7.4–12)
PCI score, median (i.q.r.)	30 (8–34)
Completeness of cytoreduction score, median (i.q.r.)	1 (0–1)
Preoperative CEA–PCI ratio, median (i.q.r.)	0.7 (0.2–4.2)
Heated intraperitoneal chemotherapy	14 (88)
Synchronous liver metastases	0 (0)
Synchronous lung metastases	0 (0)
**Hospital stay**	
Duration of ICU stay (days), median (i.q.r.)	3.8 (2.0–6.8)
Duration of hospital stay after index peritonectomy (days), median (i.q.r.)	36 (15–48)
**Histopathological data**	
Total no. of lymph nodes sampled†	10 (7–20)
No. of positive lymph nodes†	0 (0–1)
Proportion of positive lymph nodes†	0 (0–5.0)
Differentiation	
Well	5 of 14 (36)
Moderate	3 of 14 (21)
Poor	6 of 14 (43)
Adenocarcinoma	
Mucinous features	11 (69)
Goblet cell	2 (13)
Mixed	2 (13)
Histological grade	
Low	7 of 13 (54)
Intermediate	2 of 13 (15)
High	4 of 13 (31)
Signet ring cells present	0 (0)
Lymphovascular invasion	4 (25)
**Follow-up data**	
Overall deaths	1 (6.3)
Metachronous liver metastases	0 (0)
Metachronous lung metastases	0 (0)

^*^Unless otherwise indicated. †Incomplete data (over 20% missing). CA, carbohydrate antigen; CEA, carcinoembryonic antigen; PCI, Peritoneal Cancer Index. Values are *n* (%) unless otherwise indicated.

This information is novel, as it has not been demonstrated previously in patients with AA. However, this merely fills a knowledge gap in terms of implications for clinical practice, without further research. Another contributing factor, currently being explored in the authors’ institution, could explain the significant disparity in metachronous liver metastases between patients with AA and those with colorectal adenocarcinoma. In the ongoing study, the possibility of mutations in the binding region (Pro–Glu–Leu–Pro–Lys; PELPK) of the *CEACAM5* gene in patients with AA or colorectal adenocarcinoma is being explored in a comparative genetic association analysis. A study by Zimmer and Thomas^5^ in 2001 explored patients who had colorectal adenocarcinoma with raised CEA levels and found mutations in the CEA glycoprotein. In these patients, there was a lower binding affinity of CEA to the CEA receptors in liver Kupffer cells; hence, they did not seem to develop metachronous liver metastases. Theorized from this, a finding of mutations in the *CEACAM5* gene that encodes the PELPK region in patients with AA could explain the significantly lower proportion of liver metastases in such patients. The results of this study will hopefully be available in early 2024.

The authors intend to conduct an RCT in which the primary resected tumour from patients with colorectal adenocarcinoma is sent for genetic sequencing to see whether the CEA PELPK genome region is intact. The plan is to separate these patients into intervention and placebo arms in order to test a suitable monoclonal antibody that would target the CEA PELPK binding motif to decrease its binding affinity with the CEA receptor, in the hope that the patient would not go on to develop metachronous liver metastases.

## Data Availability

The data that support the findings of this study are available from the corresponding author upon reasonable request.
